# Transcriptome-wide association study identifies susceptibility genes for rheumatoid arthritis

**DOI:** 10.1186/s13075-021-02419-9

**Published:** 2021-01-22

**Authors:** Cuiyan Wu, Sijian Tan, Li Liu, Shiqiang Cheng, Peilin Li, Wenyu Li, Huan Liu, Feng’e Zhang, Sen Wang, Yujie Ning, Yan Wen, Feng Zhang

**Affiliations:** School of Public Health, Xi’an Jiaotong University Health Science Center; Key Laboratory of Trace Elements and Endemic Diseases, National Health Commission of the People’s Republic of China, No.76, Yan Ta West Road, Xi’an, 710061 People’s Republic of China

**Keywords:** Rheumatoid arthritis (RA), Transcriptome-wide association study (TWAS), Genome wide association study (GWAS), Susceptibility genes

## Abstract

**Objective:**

To identify rheumatoid arthritis (RA)-associated susceptibility genes and pathways through integrating genome-wide association study (GWAS) and gene expression profile data.

**Methods:**

A transcriptome-wide association study (TWAS) was conducted by the FUSION software for RA considering EBV-transformed lymphocytes (EL), transformed fibroblasts (TF), peripheral blood (NBL), and whole blood (YBL). GWAS summary data was driven from a large-scale GWAS, involving 5539 autoantibody-positive RA patients and 20,169 controls. The TWAS-identified genes were further validated using the mRNA expression profiles and made a functional exploration.

**Results:**

TWAS identified 692 genes with *P*_TWAS_ values < 0.05 for RA. CRIPAK (*P*^EL^ = 0.01293, *P*^TF^ = 0.00038, *P*^NBL^ = 0.02839, *P*^YBL^ = 0.0978), MUT (*P*^EL^ = 0.00377, *P*^TF^ = 0.00076, *P*^NBL^ = 0.00778, *P*^YBL^ = 0.00096), FOXRED1 (*P*^EL^ = 0.03834, *P*^TF^ = 0.01120, *P*^NBL^ = 0.01280, *P*^YBL^ = 0.00583), and EBPL (*P*^EL^ = 0.00806, *P*^TF^ = 0.03761, *P*^NBL^ = 0.03540, *P*^YBL^ = 0.04254) were collectively expressed in all the four tissues/cells. Eighteen genes, including ANXA5, AP4B1, ATIC (*P*_TWAS_ = 0.0113, downregulated expression), C12orf65, CMAH, PDHB, RUNX3 (*P*_TWAS_ = 0.0346, downregulated expression), SBF1, SH2B3, STK38, TMEM43, XPNPEP1, KIAA1530, NUFIP2, PPP2R3C, RAB24, STX6, and TLR5 (*P*_TWAS_ = 0.04665, upregulated expression), were validated with integrative analysis of TWAS and mRNA expression profiles. TWAS-identified genes functionally involved in endoplasmic reticulum organization, regulation of cytokine production, TNF signaling pathway, immune response-regulating signaling pathway, regulation of autophagy, etc.

**Conclusion:**

We identified multiple candidate genes and pathways, providing novel clues for the genetic mechanism of RA.

**Supplementary Information:**

The online version contains supplementary material available at 10.1186/s13075-021-02419-9.

## Introduction

Rheumatoid arthritis (RA) is a chronic, inflammatory, autoimmune disease primarily affecting the joints, even probably leading to accumulating joint damage and irreversible disability. Epidemiological studies in RA show that it affects up to 0.5–1% of the general adult population worldwide. Approximately two thirds of cases are characterized by rheumatoid factor or autoantibodies that target various molecules including modified self-epitopes [1]. Some strong genetic components are known to be involved in the development of RA. Twins and family studies offered a strong suggestion that the risk of RA increased in individuals with an RA family history by shared genetic factors [2–4]. The recognition of key genetic components and mechanisms will lead to a better understanding of the pathogenesis of RA.

Genome-wide association study (GWAS) represents a powerful approach of understanding the genetic basis of many complex traits in common human diseases. Especially, it was proved extremely well-suited to the identification of common single nucleotide polymorphism (SNP)-based variants with modest to large effects on phenotype. GWAS with fine mapping, candidate gene approaches, and a meta-analysis of GWAS has identified ~ 100 loci across the genome harboring RA susceptibility variants [5]. However, the specific biological mechanisms and functional consequences of many genetic variants identified by GWAS remain largely unknown, in particular, their role on disease severity; that is to say, the GWAS approach is likely to miss expression-trait associations of small effect.

Gene expression is an intermediate phenotype between genetic variant and traits underlying disease susceptibility. Many genetic variants devote their effects on complex traits by modulating gene expression [6]. Unfortunately, large-scale expression-trait associations are hampered by specimen availability and cost, as well as intrinsic factor, small effects. Consequently, transcriptome-wide association study (TWAS) was developed to address these problems, which integrates gene expression with large-scale GWAS [7]. It uses a small set of individuals with both genotype and gene expression data as a reference panel to identify significant expression-trait associations. Through extensive simulations of available GWAS data, TWAS identified candidate genes associated with, schizophrenia [8], calcific aortic valve stenosis [9], nonobstructive azoospermia [10], inflammatory biologic age [11], and other complex traits [12, 13]. Meanwhile, the genetic susceptibility variants associated with RA commonly map to enhancer regions [14], which can regulate one or more genes at distant locations in a cell-type-specific manner. Thus, the improved understanding of gene regulation relationship defining which genes are important in which cell types is vital for the predisposition to RA.

In this study, we conducted cell/tissue-related TWAS for RA based on the GWAS dataset and gene expression from EBV-transformed lymphocytes (EL), transformed fibroblasts (TF), peripheral blood (NBL), and whole blood (YBL). We subsequently reevaluated the expression of the TWAS-identified genes and made a functional exploration. This is the first time that TWAS is applied to a large-scale GWAS data to detect susceptibility genes associated with RA.

## Methods

### GWAS summary data of RA

A recent large-scale genome-wide association study meta-analysis of RA was used here [15]. Briefly, the genome-wide summary data was collected from six GWAS collections, 5539 cases and 20,169 controls in total, per-collection: Brigham Rheumatoid Arthritis Sequential Study (483 cases, 1449 controls), Canada (589 cases, 1472 controls), Epidemiological Investigation of Rheumatoid Arthritis (1173 cases, 1089 controls), North American Rheumatoid Arthritis Consortium I (867 cases, 1041 controls) and III (902 cases, 4510 controls), and Wellcome Trust Case Control Consortium (1525 cases, 10,608 controls); typed at 2,556,272 SNPs. Genotyping was conducted using commercial platforms, such as Affymetrix 6.0 array and Illumina 550 K array. RA cases either met the 1987 American College of Rheumatology criteria for the diagnosis of rheumatoid arthritis or were diagnosed by board-certified rheumatologists, with the limitation of anti-cyclic citrullinated peptide (anti-CCP) positive or rheumatoid factor (RF) positive. Detailed information of cohorts, genotyping, imputation, meta-analysis, and quality control approaches can be found in the published studies [15–20].

### TWAS

FUSION software was applied to the RA GWAS summary data for cell/tissue-related TWAS analysis, including EL, TF, NBL, and YBL. TWAS analysis used pre-computed gene expression weights together with disease GWAS summary statistics to calculate the association of every gene to disease. The genetic values of expression were computed one probe set at a time using SNP genotyping data located 500 kb on either sides of the gene boundary. For this study, the gene expression weights of EL, TF, NBL, and YBL were driven from the FUSION website (http://gusevlab.org/projects/fusion/).

### Validating TWAS results by genome-wide mRNA expression profiles of RA

The expression data of RA were downloaded from Gene Expression Omnibus (GEO) DataStets (https://www.ncbi.nlm.nih.gov/sites/GDSbrowser?acc=GDS3794) and corresponding reference [21]. In the study, a complete genome-wide transcript profiling of peripheral blood mononuclear cells (PBMCs) from 18 RA patients and 15 age- and sex-matched healthy controls were collected. The information about the demographical and clinical characteristics of RA patients and controls was presented in Table S1. The total RNA was isolated from PBMCs using the PAXgene RNA isolation kit (PreAnalytix). The concentration and profiles of total RNA were determined using a NanoDrop ND-1000 (Wilmington) and a Bioanalyzer 2100 (Agilent). Two hundred nanograms of RNA was reverse transcribed. The cDNA was transcribed and cRNA was labeled with biotin-16-UTP. Labeled probe hybridization to Illumina BeadChips human-6v2 was carried out using Illumina’s BeadChip 6v2 protocol. Beadchips were scanned on the Illumina BeadArray 500GX Reader. The preliminary data analysis were performed and normalized by Illumina BeadStudio software. Then the real-time PCR was further performed to detect mRNA expression. Differential analysis per gene was performed with one-way analysis of variance (ANOVA) and *P* values were adjusted to control the false discovery rate (FDR, 5%).

### Functional exploration

The significant genes identified by TWAS were further made a functional exploration that included Gene Ontology (GO), pathway analysis, and PPI network construction using an online analysis tool of Gene Annotation & Analysis Resource, Metascape (http://metascape.org). GO analysis was based on Fisher’s exact test and calculation of *P* values. Pathway analysis was performed for differentially expressed genes based on the database. The construction of PPI network and associated module analysis was based on GO enrichment analysis using the plugin Molecular Complex Detection (MCODE). MCODE algorithm was then applied to this network to identify neighborhoods where proteins are densely connected.

## Results

### TWAS analysis results of RA

**Fig. 1:**
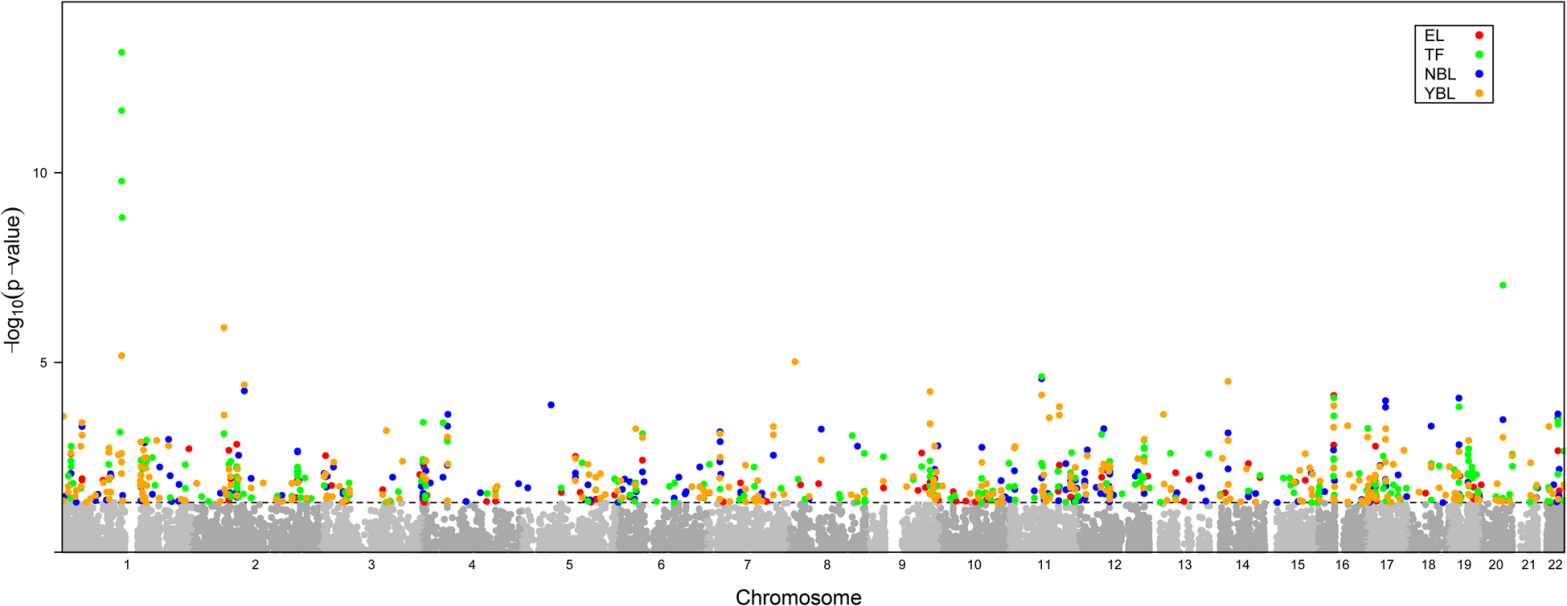
Manhattan plot of TWAS-identified genes and significantly expressed genes associated with RA (colorful points). Each point represents a single gene, with physical position (chromosome localization) plotted on the *x*-axis and -log10 (*P* value) of association between gene and RA plotted on the *y*-axis. The significant genes in different tissues/cells are highlighted with different colors (red, EL; green, TF; blu, NBL; yellow, YBL; gray, all)

**Fig. 2:**
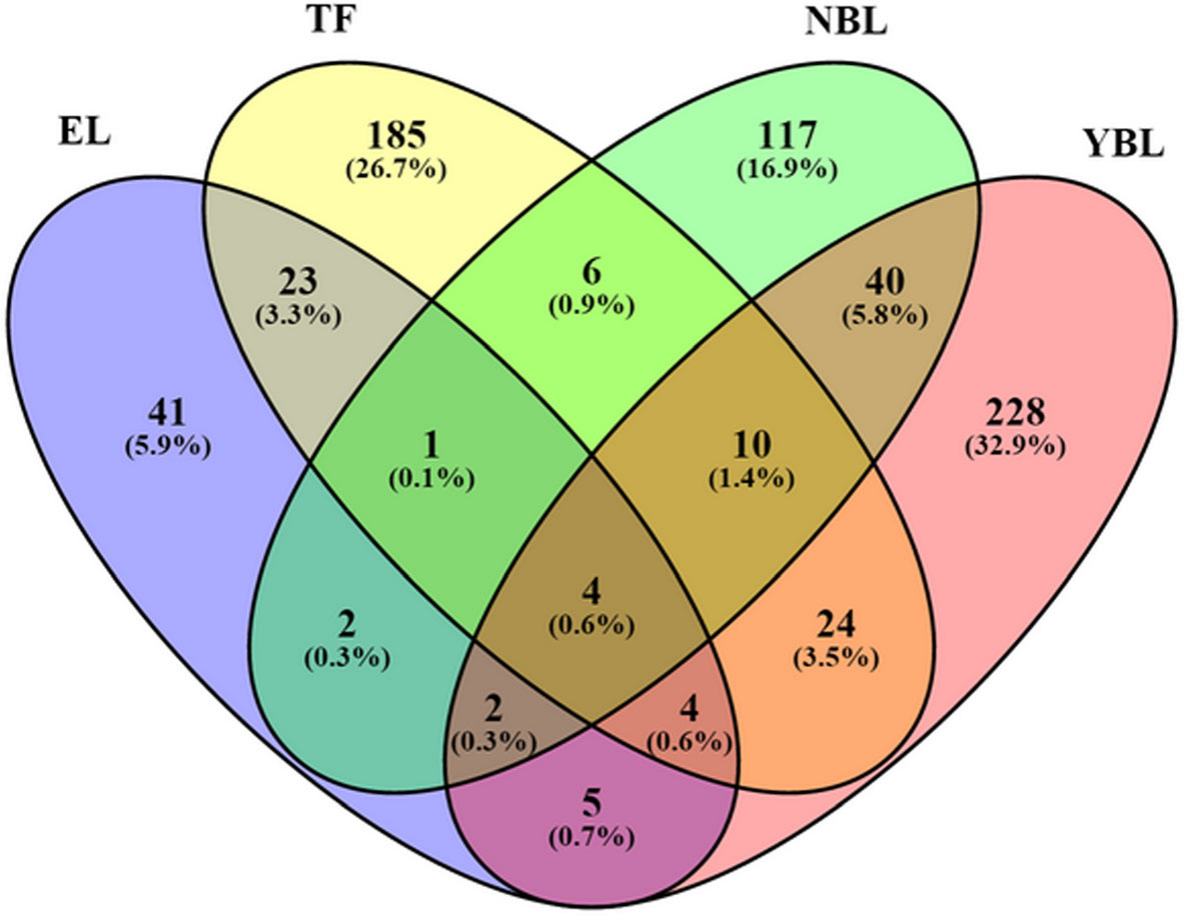
Venn diagram reveals the overlap of TWAS-significant genes in different tissues/cells. Purple, EL; yellow, TF; green, NBL; pink, YBL

Using the GWAS summary data, TWAS identified 8403 genes in total, and 1440, 4224, 2410, and 4628 genes for EL, TF, NBL, and YBL, respectively. In the gene list, there were 692 significant genes with TWAS *P* values < 0.05 in total (Fig. 1), including 82 genes for EL, 257 genes for TF, 182 genes for NBL, and 317 genes for YBL. Different tissues or cells have their own gene expression profile. In order to find out the most representative genes, we carried out an overlap analysis of genes in different tissues/cells. The Venn diagram (Fig. 2) showed that the number of genes expressed in one or more tissues/cells. For example, there were 82 TWAS-identified significant genes associated with RA in EL; there were 32 significant genes in both EL and TF; there were 5 significant genes in EL, TF, and YBL; and there were 4 significant genes in EL, TF, YBL, and NBL. The four novel TWAS-significant RA susceptibility genes identified in all four tissues/cells were CRIPAK, MUT, FOXRED1, and EBPL, which were located on chromosomes 4, 6, 11, and 13, respectively. Table 1 presented the more detailed information of the four genes, including heritability of genes (HSQ), rsID of the most significant GWAS SNP in the locus (BEST.GWAS.ID), number of SNPs in the locus (NSNP), and TWAS *P* value (*P*_TWAS_).

**Table 1:** The TWAS-significant RA susceptibility genes in all four tissues/cells

Tissue/cell	Gene	CHR	HSQ	BEST.GWAS.ID	NSNP	***P***_**TWAS**_
EL	CRIPAK	4	0.488	rs3755963	326	0.01293
EL	MUT	6	0.475	rs6458697	353	0.00377
EL	FOXRED1	11	0.604	rs602735	634	0.03834
EL	EBPL	13	0.410	rs1198329	391	0.00806
TF	CRIPAK	4	0.353	rs3755963	326	0.00038
TF	MUT	6	0.513	rs6458697	353	0.00076
TF	FOXRED1	11	0.390	rs602735	634	0.01120
TF	EBPL	13	0.287	rs1198329	391	0.03761
NBL	CRIPAK	4	0.134	rs3755963	333	0.02839
NBL	MUT	6	0.033	rs6458697	357	0.00778
NBL	FOXRED1	11	0.029	rs602735	650	0.01280
NBL	EBPL	13	0.073	rs1198329	401	0.03540
YBL	CRIPAK	4	0.345	rs3755963	301	0.00978
YBL	MUT	6	0.084	rs6458697	357	0.00096
YBL	FOXRED1	11	0.222	rs602735	641	0.00583
YBL	EBPL	13	0.521	rs1198329	400	0.04254

*CHR* chromosome, *HSQ* heritability of the gene, *BEST.GWAS.ID* rsID of the most significant GWAS SNP in locus, *NSNP* number of SNPs in the locus, *P*_TWAS_ TWAS *P* value

### Validating TWAS results by mRNA expression profiles of RA

Genome-wide transcriptional profiles of PBMCs from 18 RA patients and 15 controls measured on microarrays found 339 differently expressed genes, among which 238 were downregulated and 101 were upregulated. Comparing the TWAS results and mRNA expression profile analysis results, 18 common genes were identified as showed in Table 2, which presented the information of the genes supplied by TWAS. In the 18 genes, ANXA5 and RAB24 presented in EL and ATIC and C12orf65 presented in TF; meanwhile, the left 14 genes presented in NBL or/and YBL.

**Table 2:** The common genes identified by both TWAS and microarray expression data

Genes	CHR	HSQ	BEST.GWAS.ID	NSNP	***P***_**TWAS**_	Regulation*
ANXA5^*e*^	4	0.449	rs7691220	439	0.04665	Down
AP4B1^*y*^	1	0.092	rs2476601	463	0.00849	Down
ATIC^*t*^	2	0.335	rs4673928	473	0.01130	Down
C12orf65^*t*^	12	0.067	rs1727323	335	0.00191	Down
CMAH^*n*^	6	0.081	rs6906654	682	0.01422	Down
PDHB^*y*^	3	0.116	rs13315591	435	0.02839	Down
RUNX3^*y*^	1	0.044	rs4520361	372	0.03460	Down
SBF1^*y*^	22	0.115	rs5771362	303	0.01867	Down
SH2B3^*y*^	12	0.046	rs4346023	243	0.00345	Down
STK38^*y*^	6	0.089	rs7764323	567	0.00056	Down
TMEM43^*y*^	3	0.049	rs1106087	556	0.03760	Down
XPNPEP1^*n,y*^	10	0.016	rs10787226	321	0.04826	Down
KIAA1530^*n*^	4	0.189	rs3755963	345	0.00462	Up
NUFIP2^*n*^	17	0.018	rs2041156	345	0.00519	Up
PPP2R3C^*n,y*^	14	0.060	rs10162419	384	0.00072	Up
RAB24^*e*^	5	0.369	rs4976688	299	0.03134	Up
STX6^*y*^	1	0.103	rs1538620	464	0.00116	Up
TLR5^*n*^	4	0.449	rs7691220	439	0.04665	Up

*CHR* chromosome, *HSQ* heritability of the gene, *BEST.GWAS.ID* rsID of the most significant GWAS SNP in locus, *NSNP* number of SNPs in the locus, *P*_TWAS_ TWAS *P* value. *Presents the regulation of gene in genome-wide transcriptional profiles measured on microarrays. ^*e*, *t, n, y*^Mean the genes were identified by TWAS in EL, TF, NBL, and YBL, respectively

### Functional exploration of the TWAS-identified genes associated with RA

**Fig. 3:**
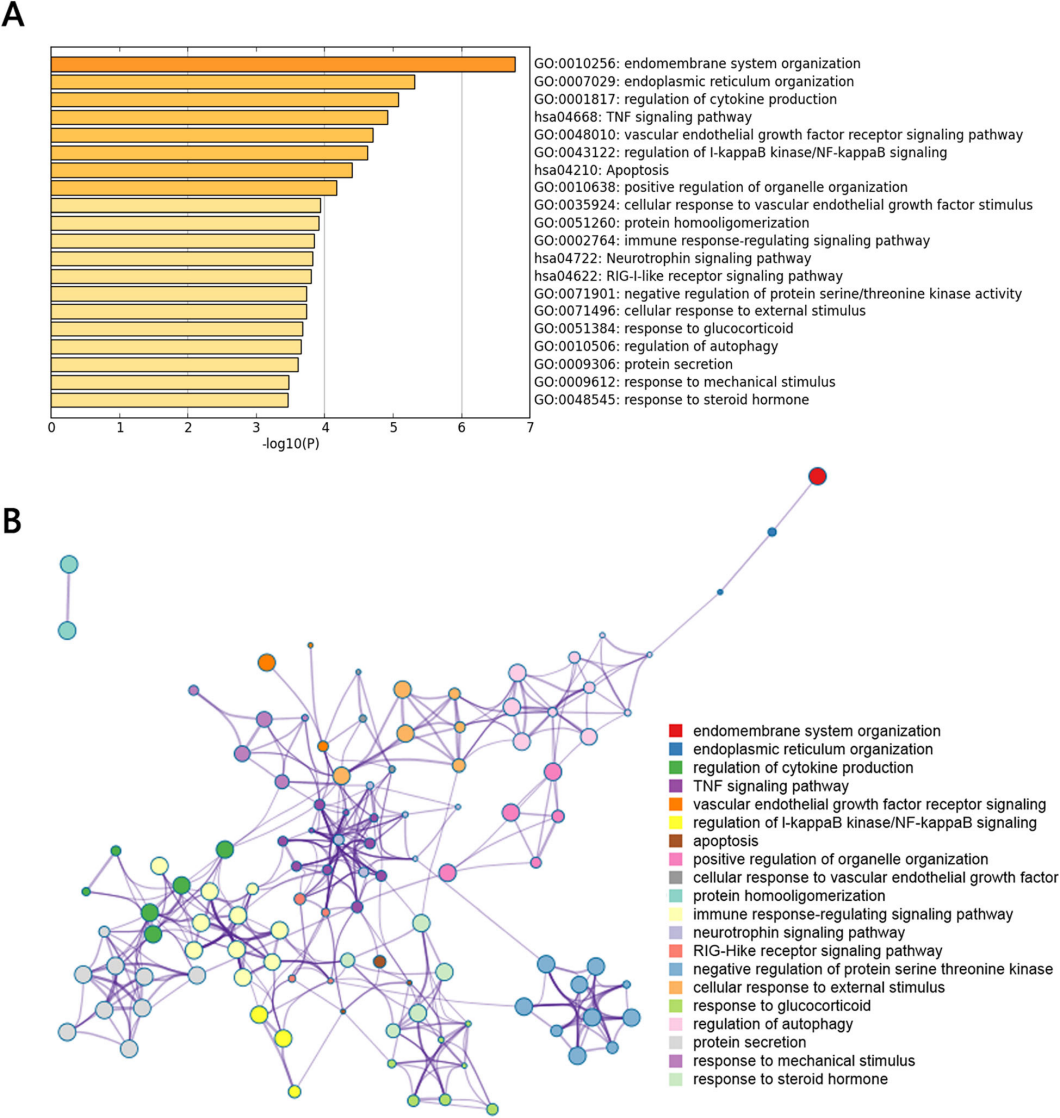
Gene ontology (GO) term analysis of differentially expressed genes. **a** Heatmap of enriched GO terms. **b** The network layout of representative GO terms under hierarchical clustering. In the network, each circle node represents a term, where its size is proportional to the number of input genes fall into that term, and its color represents its cluster identity (i.e., nodes of the same color belong to the same cluster). Terms with a similarity score > 0.3 are linked by an edge (the thickness of the edge represents the similarity score)

**Fig. 4:**
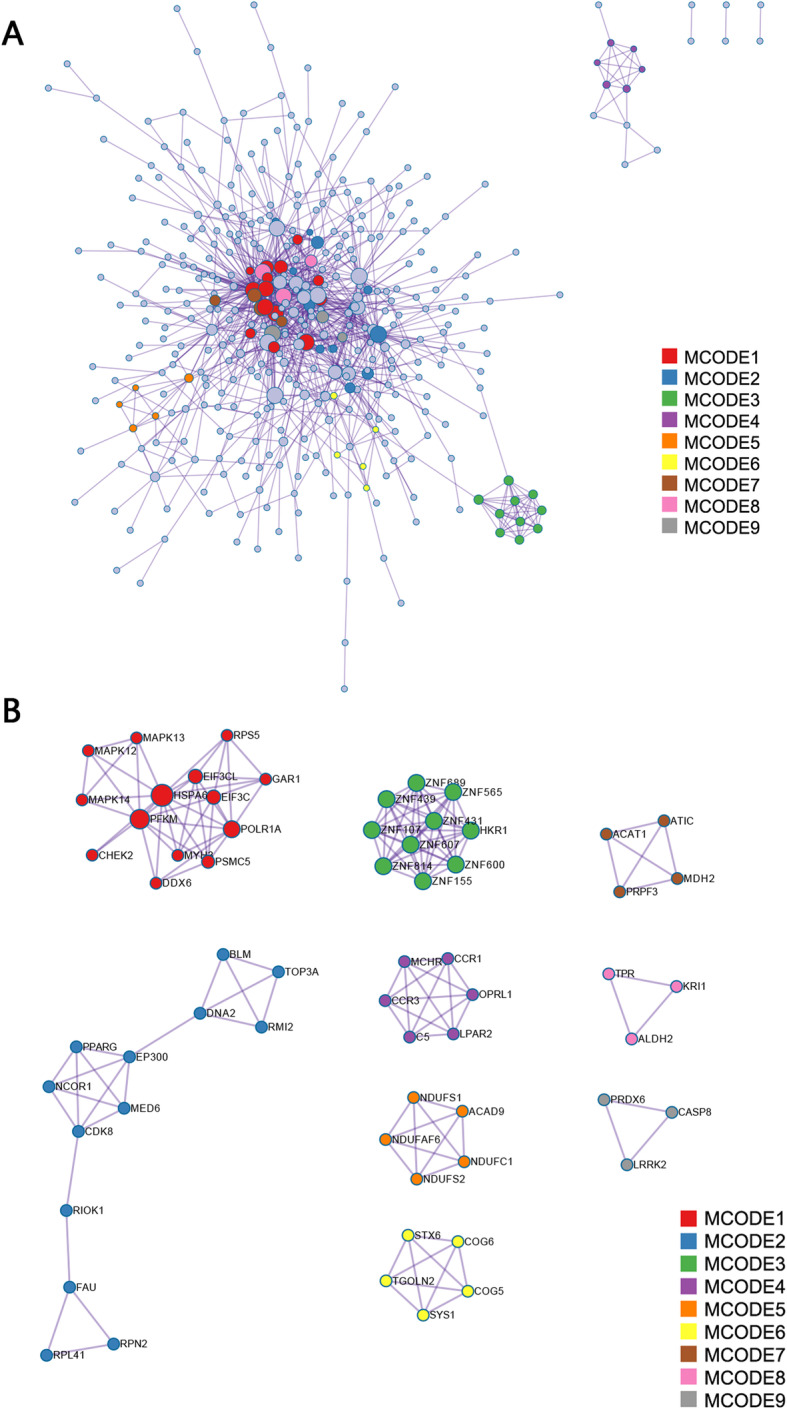
PPI network and the significant module. **a** PPI network of the TWAS-identified genes. **b** Significant modules of the PPI network

Pathway and process enrichment analysis was carried out with the following ontology sources: KEGG Pathway, GO Biological Processes, GO Molecular Functions, Reactome Gene Sets, Canonical Pathways, and CORUM. The total 654 TWAS-identified genes in the four tissues/cells were successfully submitted to Metascape performing GO enrichment analysis. TWAS-identified genes were annotated with an enrichment of biological processes and KEGG pathways involved in endomembrane system organization, endoplasmic reticulum organization, regulation of cytokine production, TNF signaling pathway, and so on (Fig. 3a). The significant terms were then hierarchically clustered, selected a subset of representative terms, and converted them into a network layout (Fig. 3b). Combining the results of Fig. 3a and b, the identified biological pathways with most significance were regulation of cytokine production, TNF signaling pathway, vascular endothelial growth factor (VEGF) receptor signaling pathway, immune response-regulating signaling pathway, regulation of autophagy, and negative regulation of protein serine/threonine kinase. A protein-protein interaction (PPI) network of the TWAS-identified genes was constructed, and module analysis was conducted using the plugin Molecular Complex Detection (MCODE). The PPI network was constructed based on 1122 GO terms (Fig. 4a). The top three GO terms were regulation of TP53 activity through phosphorylation, retrograde transport at the trans-golgi-network, and regulation of TP53 activity. The significant modules from the PPI network formed 9 MCODE clusters with a class of genes (Fig. 4b), for example, MCODE1, MCODE3, and MCODE5 were characterized by MAPK (mitogen-activated kinase-like protein) family genes, ZNF (zinc finger protein) family genes, and NDUF (NADH ubiquinone oxidoreductase subunit) family genes, respectively.

## Discussion

TWAS is a creative and valuable analysis method that can integrate genetic variation with gene expression to identify genes whose cis-regulated expression is associated with complex traits. It captures heterogeneous signals better than individual SNPs or cis-eQTLs and focuses prediction on the genetic component of expression that avoids confounding from environmental differences caused by the trait that may influence expression. What is more, TWAS avoids tissue acquisition challenges that may pose the greatest hurdle for producing larger datasets. Thus, TWAS has been widely applied to yield mechanistic disease insights, yet the first time for RA in this study.

RA is a systemic disease and a variety of immunological events occur not only in joints but also outside the joint at mucosal surfaces and primary lymphoid tissues, especially synovium. Thus, various types of tissues and cells will be attacked by the disease, including the synovium, cartilage, bone, fibroblasts, adipocytes, macrophage, immune cells, and so on. In this work, we conducted cell/tissue-related TWAS for RA. TWAS identified a total of 674 genes with transcriptome-wide-significant associations with RA in four tissues/cells. CRIPAK, MUT, FOXRED1, and EBPL, which were collectively expressed in all the four tissues/cells, were novel genes associating with RA. Consistent with the result of TWAS, eighteen genes, ANXA5, AP4B1, ATIC, C12orf65, CMAH, PDHB, RUNX3, SBF1, SH2B3, STK38, TMEM43, XPNPEP1, KIAA1530, NUFIP2, PPP2R3C, RAB24, STX6, and TLR5, have been reported differently expressed in peripheral blood mononuclear cells of RA patients.

Cysteine-rich PAK1 inhibitor (CRIPAK) is an endogenous inhibitor of p21-activated protein kinase 1 (PAK1), which interacts with Pak1 through the N-terminal regulatory and inhibited the enhancement of estrogen receptor transactivation. The decrease in gene expression of CRIPAK could act to promote the accumulation of phosphorylated myosin light chain and its stimulation of actomyosin ATPase activity in laser-captured serotonin neurons from macaques treated with ovarian hormones [22]. There are few reports about the role and mechanism of CRIPAK in diseases, especially no reports in RA. However, PAK1 has been extensively studied. PAK1, a potential mediator of Rac1/Cdc42 signaling pathway, is involved in regulating the migration, invasion, proliferation, and inflammation of fibroblast-like synoviocytes from rheumatoid arthritis patients [23, 24]. These studies indirectly support the potential role of CRIPAK in rheumatoid arthritis.

Methylmalonyl-CoA mutase (MUT) encodes the mitochondrial enzyme methylmalonyl coenzyme A mutase. In humans, the gene encoded enzyme catalyzes the isomerization of methylmalonyl-CoA to succinyl-CoA, while this enzyme may have different functions in other species. FAD-dependent oxidoreductase domain containing 1 (FOXRED1) encoded protein that is localized to the mitochondria and whose function is involved in assembly, stability, and/or correct functioning of complex I [25, 26]. Numerous processes involved in mitochondrial function are related to RA. For example, oxidative stress impairs energy metabolism in primary cells and synovial tissue of RA patients [27]; the interaction of abnormal cellular metabolism, mitochondrial dysfunction, hypoxia, and the proinflammatory signaling pathways in synovial cells is contributed to synovial invasiveness of RA [28]; and rare/low-frequency variants of the mitochondria respiratory chain-related proteins were aggregated RA patients [29]. Meanwhile, The top three GO terms in MCODE 5 of PPI network were complex I biogenesis, NADH dehydrogenase complex assembly, and mitochondrial respiratory chain complex I assembly. Therefore, we predict the two genes may play roles in the pathology of RA via affecting mitochondrial function.

EBPL is an emopamil-binding protein (EBP)-like protein. EBP is a high-affinity binding protein for [H-3] emopamil and belongs to the family of so-called sigma receptors. Mutations disrupted EBP impair cholesterol biosynthesis and cause X-chromosomal dominant chondrodysplasia punctate. The EBPL mRNA was expressed ubiquitously and most abundant in the liver, lung, and kidney. However, EBPL has a yet-to-be-discovered function [30].

In the common RA-associated gene list identified by TWAS, we should pay attention to ATIC, RUNX3, and TLR5. 5-Aminoimidazole-4-carboxamide ribonucleotide formyltransferase/IMP cyclohydrolase (ATIC) encodes a bifunctional protein, which catalyzes the last two steps of the de novo purine biosynthetic pathway. ATIC plays a crucial role in the mechanisms underlying methotrexate’s anti-inflammatory and antiproliferative effects. ATIC missense variant and gene polymorphism affects response to methotrexate treatment in RA patients [31–33]. Runt-related transcription factor 3 (RUNX3) encodes a member of the runt domain-containing family of transcription factors and involves on T cell development, T cells polarization, and T cell selection [34, 35]. RA is characterized by the presence of activated T lymphocytes. It is indicated that RUNX3 may play roles on the mechanisms of T cell activation in RA. Toll-like receptor 5 (TLR5) encodes a member of the TLR family that plays an essential role in pathogen recognition and innate immune response activation. TLR5 agonist, flagellin, can promote monocyte infiltration and osteoclast maturation directly through myeloid TLR5 ligation and indirectly via TNF-alpha production from RA and mouse cells [36]. Angiogenesis in RA is fostered directly by TLR5 ligation and indirectly through interleukin-17 induction [37]. TLR5 is the bridge that interconnects the formation of new blood vessels with the maturation of joint osteoclasts, thereby accelerating the bone destruction process in RA [38]. There have been other researches indicating that TLR5 is involved on RA inflammation, bone destruction, and angiogenesis; thus, TLR5 is a critical element and target for RA mechanism.

In the identified biological pathway, TNF signaling pathway, and regulation of autophagy become areas of our interest. The role of TNF signaling pathway in the pathogenesis and progression of RA is obviously important. The approach of targeting TNF has become a considerably effective treatment of RA [39]. It would be of great significance for the prevention and treatment of RA to establish a genetic link between gene expression and RA susceptibility. In addition, in the results presented in Fig. 3b, TNF signaling pathway is associated with several pathways, such as VEGF receptor signaling, cellular responses to external stimulus, immune response-regulating signaling pathway, and regulation of cytokine production, which suggested a possible new perspective that different cell functions are linked by genetic inheritance and then affect the occurrence, development, and treatment of RA. Autophagy is another biological function that plays pivotal roles in RA. It has been evident that dysregulated autophagy damages the maturation survival and proliferation of various immune and non-immune cells in RA pathogenesis, as well as affects the antigen presentation of immune cells [40]. Distinguishing the genes’ polymorphisms in autophagy or other cellular mechanisms in RA pathology is of great significance in establishing the association of autophagy with RA.

There are two limitations of the study that needed attention. First, despite TWAS has great power and is not confounded by reverse causality (disease → gene expression independent of SNP), instances of pleiotropy (multiple effects by a single SNP or linked SNPs involved on gene expression and RA) are statistically indistinguishable from real susceptibility genes with causal link. Second, this study did not include experimental analysis based on RA patients with different stages, different therapies, and so on, which will be considered in future studies.

With TWAS in this study, we found that the mRNA expression of some genes in human tissues/cells can be affected by SNPs, further associating with RA susceptibility. For example, four genes (CRIPAK, MUT, FOXRED1, and EBPL) in four distinct loci (rs3755963, rs6458697, rs602735, and rs1198329) were associated with RA susceptibility. We carried out a TWAS strategy to pinpoint RA-associated genes; both genomics and transcriptomics were combined, and cis-heritable genes were explored and evaluated efficiently. The study provides a potential functional mechanism of how genetic variants on chromosome may increase RA susceptibility.

## Conclusions

In summary, the TWAS study identifies novel and common susceptibility genes for rheumatoid arthritis. Beyond specific mechanistic findings for RA, this work outlines a systematic approach to identify functional mediators of complex disease.

## Supplementary Information

**Additional file 1.**

## Data Availability

The gene expression datasets generated during the study are available in the FUSION website (http://gusevlab.org/projects/fusion/).

## Abbreviations

RA: Rheumatoid arthritis
GWAS: Genome-wide association study
TWAS: Transcriptome-wide association study
EL: EBV-transformed lymphocytes
TF: Transformed fibroblasts
NBL: Peripheral blood
YBL: Whole blood
SNPs: Single nucleotide polymorphisms
Anti-CCP: Anti-cyclic citrullinated peptide
RF: Rheumatoid factor
GEO: Gene expression omnibus
PBMCs: Peripheral blood mononuclear cells
ANOVA: Analysis of variance
FDR: False discovery rate
GO: Gene ontology
MCODE: Molecular complex detection
CHR: Chromosome
HSQ: Heritability of the gene
BEST.GWAS.ID: rsID of the most significant GWAS SNP in locus
NSNP: Number of SNPs in the locus
*P*_TWAS_: TWAS *P* value
VEGF: Vascular endothelial growth factor
PPI: Protein-protein interaction
MAPK: Mitogen-activated kinase-like protein
ZNF: Zinc finger protein
NDUF: NADH ubiquinone oxidoreductase subunit
CRIPAK: Cysteine-rich PAK1 inhibitor
PAK1: P21-activated protein kinase 1
MUT: Methylmalonyl-CoA mutase
FOXRED1: Oxidoreductase domain containing 1
EBP: Emopamil-binding protein
RUNX3: Transcription factor 3
TLR5: Toll-like receptor 5
