# Supplementary material for: Transcriptome-wide association study identifies susceptibility genes for rheumatoid arthritis
Source: Arthritis Res Ther. 2021 Jan 22;23:38. doi: 10.1186/s13075-021-02419-9 (PMC7821659; doi:10.1186/s13075-021-02419-9)
Supplement: Supplementary file 1 — Additional file 1. [file 13075_2021_2419_MOESM1_ESM.docx]

Table S1. Demographic and clinical characteristics of the RA patients and controls.

| Characteristic index | RA patients | Controls |
| --- | --- | --- |
| n | 18 | 15 |
| Mean age (years) | 60 | 57 |
| Women (%) | 72.3 | 73.3 |
| Caucasian (%) | 100 | 100 |
| RF-positive (%) | 88.9 | / |
| ACPA- positive (%) | 90.9 (out of 11 RA patients) | / |
| Mean disease duration (years) | 8.6 | / |
| Erosions (%) | 63.2 | / |
| Disease Activity Score 28 (DAS28) mean | 5.22 | / |
| Disease-Modifying Anti-Rheumatic Drugs use (%) | 100 | / |
| Anti-TNF therapy | 0 | / |

Note: RF, rheumatoid factor; ACPA, anti-cyclic citrullinated peptide antibodies.
